# Speed Biases With Real-Life Video Clips

**DOI:** 10.3389/fnint.2018.00011

**Published:** 2018-03-16

**Authors:** Federica Rossi, Elisa Montanaro, Claudio de’Sperati

**Affiliations:** ^1^Laboratory of Action, Perception and Cognition, Faculty of Psychology, Vita-Salute San Raffaele University, Milan, Italy; ^2^Department of Neuroscience Rita Levi Montalcini, University of Turin, Turin, Italy; ^3^Experimental Psychology Unit, Division of Neuroscience, San Raffaele Scientific Institute, Milan, Italy

**Keywords:** speed biases, motion perception, reality monitoring, video clips, intuitive physics

## Abstract

We live almost literally immersed in an artificial visual world, especially motion pictures. In this exploratory study, we asked whether the best speed for reproducing a video is its original, shooting speed. By using adjustment and double staircase methods, we examined speed biases in viewing real-life video clips in three experiments, and assessed their robustness by manipulating visual and auditory factors. With the tested stimuli (short clips of human motion, mixed human-physical motion, physical motion and ego-motion), speed underestimation was the rule rather than the exception, although it depended largely on clip content, ranging on average from 2% (ego-motion) to 32% (physical motion). Manipulating display size or adding arbitrary soundtracks did not modify these speed biases. Estimated speed was not correlated with estimated duration of these same video clips. These results indicate that the sense of speed for real-life video clips can be systematically biased, independently of the impression of elapsed time. Measuring subjective visual *tempo* may integrate traditional methods that assess time perception: speed biases may be exploited to develop a simple, objective test of reality flow, to be used for example in clinical and developmental contexts. From the perspective of video media, measuring speed biases may help to optimize video reproduction speed and validate “natural” video compression techniques based on sub-threshold temporal squeezing.

## Introduction

Motion pictures—videos—are becoming pervasive in our everyday life. Yet, videos might easily fool us. For example, we recently showed that observers fail to notice large speed manipulations when viewing a soccer match video clip (de’Sperati and Baud Bovy, 2017). In that study, we also found a small but reliable tendency toward speed underestimation, which suggests that the best speed for reproducing a video may not be its original shooting speed. This may sound rather counterintuitive, as we tend to implicitly assume that shooting speed and reproduction speed should coincide, for otherwise motion rendering would be sub-optimal or even artifactual^1^. Yet, this may not always be true.

There are several reasons why real-life motion pictures could generate a “wrong” speed impression, or speed bias. In general, this happens whenever the scene does not match expectations, either implicit or explicit, about how the world should appear (Shi et al., 2013; Shi and Burr, 2016). Scene content and context may induce the viewer to expect that events should unfold at a different pace, for example when an action is performed at a particularly slow rhythm or when motion cues are poor. In this respect, there are virtually no limits as to the potential mismatches between expectations and particular visual scenes. Sometimes the wrong expectations apply even to basic physical facts (intuitive physics, McCloskey and Kohl, 1983; McCloskey et al., 1983; Pittenger, 1985; Kaiser et al., 1992; Kubricht et al., 2017). Conversely, we may be particularly well tuned to certain patterns of biological motion, either based on purely visual mechanisms or through visuo-motor coupling (de’Sperati and Stucchi, 1995, 1997, 2000; Viviani et al., 1997; de’Sperati and Viviani, 1997; Thornton, 1998; Runeson et al., 2000; Cattaneo and Rizzolatti, 2009; Gallese et al., 2009; Lacquaniti et al., 2014). Low-level factors are also important in interpreting visual motion. For example, depending on spatial and temporal frequency content, image contrast can introduce distortions in perceived speed (Anstis, 2003; Burr and Thompson, 2011). The frequently reported speed underestimation at low contrast can be an effect of different weights of low-pass and band-pass cortical filtering (Thompson et al., 2006) or as a consequence of a low-speed prior (Weiss et al., 2002). Thus, video clips depicting different real-life scenes might produce different speed biases for a variety of reasons. The first aim of this study is to verify whether this is indeed the case.

Viewing conditions could also modify the impression of speed. For example, if scaling mechanisms for speed constancy (McKee and Smallman, 1998; Distler et al., 2000; Thornton et al., 2014) do not fully compensate for display size or viewing distance, watching a video on a mobile phone may be different from watching it on a computer monitor or home TV. Likewise, watching a muted video may be different from watching it with an accompanying soundtrack, and in turn the type of soundtrack can convey a feeling of urgency or relaxation, possibly impacting on the impression of visual *tempo* (Recanzone, 2003; Soto-Faraco and Väljamäe, 2012). Indeed, there is ample evidence that music can influence the sense of time, and specific neural correlates have been proposed (Schäfer et al., 2013). Moreover, music, rhythm and movements are tightly intertwined, and this relationship extends to visual metrical perception, including a specific effect of visual motion on auditory *tempo* (Su and Jonikaitis, 2011; Su and Salazar-López, 2016). Thus, speed biases may depend on these “accessory” factors as well. Playing video clips at a slightly different speed might compensate for these effects, if present—think for example of an automatic equalization system for compensating different perceived speeds on small mobile screens vs. TV screens. The second aim of this study is to verify whether screen size or soundtrack can modify the dynamic appearance of videos.

The impression that a given visual scene is too slow—speed underestimation—may depend on the fact that its duration is perceived to be too long (and vice-versa). One reason could be that, subjectively, the scene is not “filling time” sufficiently. Indeed, according to an influential model based on the idea that temporal cognition depends on the accumulation of event ticks, perceived time is dilated when a visual stimulus “fills” it more densely, for example because of a higher speed or higher temporal frequency (Brown, 1995; Block and Zakay, 1997; Lacquaniti et al., 2014). Thus, it is possible that speed underestimation is associated to duration overestimation, although with complex motion scenes additional factors may influence time perception, especially when human actions are involved (Grivel et al., 2011; Carrozzo and Lacquaniti, 2013; Sgouramani and Vatakis, 2014). Alternatively, the lack of correlation between speed bias and duration estimation would suggest that measuring the sense of speed is not just another way to measure perceived elapsed time, but characterizes a distinct function at the interface between perception and cognition. The third aim of this study is to verify whether perceived speed and perceived duration of real-life video clips are correlated.

To address these three issues, we assessed the subjectively estimated “natural” speed of short video clips depicting various real-life scenes. This was achieved by measuring the point of subjective equality (PSE; Ehrenstein and Ehrenstein, 1999), which provided an estimate of speed bias. We then searched for a correlation between speed bias and errors in estimating temporal intervals, evaluated through a duration reproduction task (Grondin, 2010), and tested the robustness of speed bias by manipulating display size and soundtrack.

## Experiment 1

This experiment assessed the ability to estimate the “natural” reproduction speed of real-life video clips, i.e., the original shooting speed, as a function of visual content. Observers adjusted the video speed in real time until a point at which speed was not perceived as too high or too low (PSE). To mimic real-life viewing conditions, no stimulus standard was provided for comparison, so that observers had to rely entirely on their internal expectations. In Bayesian terms, this amounts to emphasizing the role of priors in perceptual decisions (“reference memory”, Shi et al., 2013). We investigated the effects of clip type, display size and repeated presentations on PSE. The same subjects were also tested in a duration reproduction task based on the same video material.

### Methods

#### Participants

Fifteen participants (mean age = 30.00 years, nine females) volunteered for the experiments. They had normal or corrected-to-normal vision, and were naïve as to the purpose of the experiment. This study was carried out in accordance with the recommendations of San Raffaele Ethical Committee. The protocol was approved by the San Raffaele Ethical Committee. All subjects gave written informed consent in accordance with the Declaration of Helsinki.

#### Stimuli and Task

We used four short video clips (duration, 30 s), displaying physical motion, human motion—first-person and third-person perspectives—and mixed human-physical motion. Three videos were shot in-house with a smartphone in HD format (30 fps, 1280 × 720 pixels, with a f/2.6 lens and 60° FOV), and one video was obtained, with permission, from a web collection of naturalistic landscapes, again in HD format^2^. Video clips were shot in fixed-camera mode^1^, except for the video clip in the first-person perspective (ego-motion). Temporal calibration was performed by recording a visual stimulus flashing at 1 Hz for 60 s. The recorded flash frequency turned out to be 0.999 Hz, which corresponds to an error of 0.1%.

Video clips represented rather uniform scenes and were displayed on a black background. C1 (jumping man—only human motion) is a frontal shot of a young man jumping in front of a building wall. C2 (foot dribbling—mixed human-physical motion) shows the same man dribbling a soccer ball in front of the same wall. C3 (water waves—physical motion) is a wide shot of an undertow of the sea, with the seashore in the front, surrounded by a few rocks, and the sea in the background. C4 (ego-motion) is a first-person perspective of a walk in a crowded street. The original video clips are available on request from the corresponding author.

Video clips were displayed on a 21″ LCD monitor at a 60 Hz refresh rate and a viewing distance of about 60 cm. We distinguish between refresh rate, i.e., the frequency at which the visual display visual buffer is updated, and frame rate, i.e., the frequency at which *different* frames are displayed. The frame rate cannot be higher than the refresh rate, but the refresh rate can be higher than the frame rate. In order to reproduce the video clips at a variable speed, participants changed the actual frame rate by means of two keyboard keys (speed increase and decrease). Given the 60 Hz refresh rate and the 30 Hz video original frame rate, reproducing a video at its original speed means displaying the same frame twice in two subsequent refresh cycles (2:1 ratio). Doubling the original video speed means displaying a video frame every refresh cycle (1:1 ratio), while halving it means displaying one video frame for four refresh cycles (4:1 ratio). Intermediate speeds are achieved by implementing appropriate ratios between the frame rate and the refresh rate. This solution for a variable speed video reproduction avoids two visual artifacts, namely, video tearing, which would result from the crude disabling of the V-synch signal, and unnatural motion, which could result from frame resampling/interpolation. In the debriefing, observers did not report motion irregularities, and videos appeared smooth. Programs were written in Matlab using the Psychophysics Toolbox extensions, and were run under Windows 7 on an Intel-based PC with on-board graphics. In this experiment videos were muted.

In this and our previous study (de’Sperati and Baud Bovy, 2017), we refer to video clip speed although technically we did not implement a gradual speed change but only discrete removal or insertion of single video frames at proper time positions. That this choice was meant to prevent video quality deterioration, as a gradual frame rate change obtained by disabling synchronization with vertical retrace signal could introduce tearing, and interpolation could generate the impression of unnatural motion. What in fact legitimates the use of the term “speed” rather than “time jumps” or the like, is the observers’ subjective impression of smooth scenes unfolding at all tested speeds, at least in our experimental conditions. Arguably, this smoothness sensation depends on the temporal integration of global motion (Burr and Santoro, 2001; Vaina et al., 2003).

#### Speed Estimation (Adjustment Task)

Observers were presented the video clips with a randomized initial speed (frame rate range: 15–60 fps). Their task was to adjust the speed by means of two keyboard keys (in 0.1% steps) in order to reach the speed that they reputed to be the original shooting speed, at which time they could skip to the next trial. Each video clip lasted 30 s, and this was also the maximum time available for speed adjustment, after which the program passed automatically to the next trial. Observers were never shown the videos at the original speed as the standard for comparison, and were instructed to be as natural as possible in trying to re-establish the original video speed, as if they were trying to fix the reproduction speed of their old, non-calibrated videotape player. A few familiarization trials with similar video clips preceded the beginning of the experimental session.

#### Duration Estimation (Interval Reproduction Task)

The same participants were also tested for their ability to reproduce the duration of video clip pieces by means of a prospective interval reproduction task (Grondin, 2010), which was administered after the adjustment task. Observers were shown short pieces of the same four video clips used in the adjustment task for a variable duration randomly extracted from a uniform distribution in the 0.5–5.5 s range, randomly intermingled, starting at a random clip position (five different durations, for a total of 60 trials for each subject). At the end, observers had to reproduce the clip piece duration by holding a keyboard key for the same amount of time.

#### Experimental Design and Data Analysis

In this experiment we tested four video clips (C1, human motion; C2, human-physical motion; C3, physical motion; C4, ego-motion), three clip sizes (21″, 10.5″ and 5.25″), and two repetitions (blocked), for a total of 24 trials for each participant. All factors were within-subject, and their presentation order was randomized within each block.

For the statistical analyses, we used both means and medians as estimators of central tendency, together with 95% bootstrap confidence intervals (method: bias corrected and accelerated percentile with 1000 runs). Normality assumption was checked with the Shapiro-Wilk test, and outliers were detected with the Grubbs test. Repeated-measures ANOVAs were used, applying the Greenhouse correction whenever necessary, in which case the degrees of freedom were non-integer. Null-hypothesis was rejected at *α* < 0.05, while in pairwise comparisons we used *α* < 0.01 to reduce multiple comparison effects. Effect size was assessed through partial eta square (*η*^2^), while associations were tested with Pearson’s *r* or Spearman’s rho according to their measurement scales. For the duration reproduction task, linear fitting of perceived vs. objective durations was performed through robust linear least-squares method.

### Results

For technical problems, three participants were excluded from the analyses, as their recordings were corrupted. Thus, in the following we report data from 12 participants. Four outlier values (1% of the trials) were replaced by mean values.

**Figure 1:**
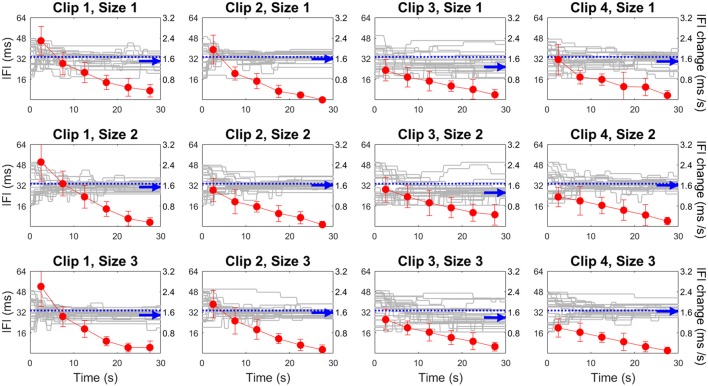
Adjustment behavior in Experiment 1. Video speed adjustments are represented through instantaneous Inter-Frame Interval (IFI, gray traces) over time in each individual trial, for each clip (columns) and display size (rows). Blue arrows indicate the mean final IFI value, which is the inverse of point of subjective equality (PSE). Red curves represent the mean adjustment rate (IFI change) over time with 95% confidence intervals. The horizontal blue dotted line indicates the original IFI of the videos (33 ms).

As an initial step, we considered the adjustment history for all participants in all trials (Figure 1). The plots illustrate the instantaneous frame rate (blue traces), expressed as the delay between two video frames (Inter-Frame Interval, IFI). IFI changed upon observer’s key strokes, starting with an initial random value. In general, participants reported feeling comfortable with the task, and they converged rather regularly towards the final estimated speed. The overall adjustment behavior is plotted as the mean IFI change (mean adjustment rate) over successive 5-s time intervals (red curves). We preferred this quantity instead of the number of key strokes because we noted that, to adjust speed, observers tended to keep holding the keyboard keys instead of pressing them frequently and briefly. Thus, the number of key strokes was not a faithful index of effective adjustment. As is evident from the figure, adjustments decreased rather regularly over time, to reach values close to zero towards the end of the trial. This reassures that trials did not end while observers were still adjusting. The mean adjustment rates over the entire trial duration were quite similar across display sizes and repetitions, the only statistically significant effect being the video clip main factor (*F*_(3,33)_ = 5.680, *p* = 0.003, *η*^2^ = 0.341).

**Figure 2:**
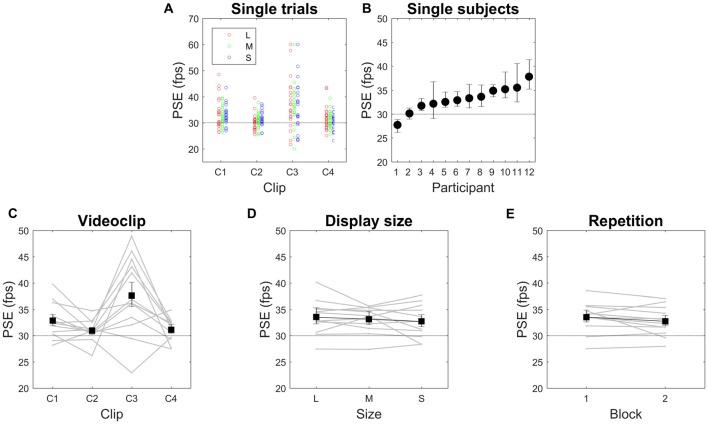
Point of subjective equality (PSE) in Experiment 1. **(A)** Single-trial data. **(B)** Single-subject data. **(C–E)** Effects of the three tested factors. In **(B–E)**, means and 95% confidence intervals are reported. Gray lines in **(C–E)** are individual subjects’ data. PSE is expressed in frame per second (fps). The horizontal dotted line indicates the original video speed (30 fps). L, M and S stand for Large, Medium and Small display size (21″, 10.5″ and 5.25″ respectively).

The final value of the adjusted speed reached at the end of the trial is the PSE, expressed as a frame rate value, which we took as a measure of speed judgment (PSE = final IFI^−1^). Figure 2A shows the PSE values measured in individual trials, separately for clip type (horizontal axis) and display size (color), while Figure 2B shows the average PSE values for each subject. Because no statistically significant interactions were found, in Figures 2C–E we then plotted the mean values and confidence intervals separately for each factor (i.e., video clip, display size and repetition), superimposed to the mean values of individual observers (gray curves). The only significant factor found to affect estimated speed was the video clip type (main effect of video clip, *F*_(1.337,14.707)_ = 7.009, *p* = 0.013, *η*^2^ = 0.389). Pairwise contrasts showed only one non-significant comparison, namely, C2 vs. C4. Despite the non-significant interactions, to further examine whether speed judgments are indeed insensitive to display size, we ran four separate ANOVAs, one for each video clip data. Again, no statistically significant effects of display size were found, except for an interaction display size × repetition with C4 (*F*_(2,22)_ = 5.305, *p* = 0.018, *η*^2^ = 0.325).

These speed judgments indicated a tendency toward speed underestimation. Indeed, the mean values of speed increase were higher than the objective video clip frame rate (30 fps) by 9%, 3%, 25% and 4% for C1, C2, C3 and C4, respectively (Figure 2C), and the lower limits of confidence intervals did not cross the 30-fps level, except in one case (C2). Similarly, by considering single subjects, in nine of them (75%) the PSEs were significantly beyond the objective video clip frame rate, again as shown by means and confidence intervals, and only in one subject was PSE significantly smaller than 30 fps (Figure 2B). The pattern of results was practically identical when computing median values (7%, 3%, 23% and 4% for C1, C2, C3 and C4, respectively, where only C2 was not significantly different from the 30-fps reference value), and also after logarithmic data transformation (geometric means: 9%, 3%, 21%, and 5% for C1, C2, C3 and C4, respectively, where only C2 was not significantly different from the 30-fps reference value). These results indicate that observers tended to judge the original video clip speed to be too low. Note that the single distributions were far from being uniform (Figure 2A), and rather tended to be slightly leptokurtic (mean kurtosis index = 0.823), that is, observers did show a speed preference.

We checked for a possible correlation between the randomly assigned initial video clip speed and the final observer’s estimation, but the two variables were not correlated at the trial-wise level (*r* = −0.010, *p* = 0.866). By contrast, we found a significant trial-wise correlation between total adjustment behavior, computed as the sum of instantaneous IFI changes produced in each trial, and speed estimation (*r* = 0.211, *p* < 0.001), which suggests that the more observers adjust, the more they underestimate video speed, regardless of the initial speed.

**Figure 3:**
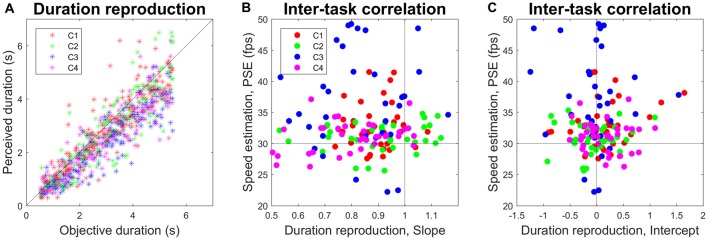
**(A)** Correlation between objective and perceived video clip duration. Each dot represents a trial. The oblique dotted line represents unitary slope with no offset. **(B)** Lack of correlation between speed estimation and duration reproduction, as measured through slope. Each dot (*N* = 144) represents, for each subject, clip and display size, the slope computed over five trials with different stimulus durations, and the PSE computed as the mean of the two repetitions in the speed estimation task. The horizontal dotted line divides the speed underestimation region (upward) from the speed overestimation region (downward). The vertical dotted line divides the time underestimation region (leftward) from the time overestimation region (rightward). **(C)** Same as **(B)** but for intercept. Colors code video clip type (C1–C4).

Figure 3 reports the results of the duration reproduction task, with the estimated video clip durations plotted against their objective durations. Reproduced durations tended to be shorter than original durations, although this effect seems to be more pronounced at longer stimulus durations. For each clip type and display size we computed a slope, which is an index of temporal overestimation (>1) or underestimation (<1). Across subjects, the mean slope was 0.89 (range = 0.81–0.93 across subjects) and was significantly less than the unitary slope (*t*_(11)_ = −7.869, *p* < 0.001). At variance with PSE in the speed estimation task, it did not depend on the video clip type (*F*_(333)_ = 2.410, *p* = 0.085, *η*^2^ = 0.180). Indeed, slope was not correlated with PSE, either subject-wise (accounted variance = 4%, *p* = 0.537, *N* = 12), subject-and-clip-wise (accounted variance = 3%, *p* = 0.222, *N* = 48), or subject-and-clip-and-size-wise (accounted variance = 2%, *p* = 0.085, *N* = 144). The intercept, which could reflect very short-range temporal processing, and was on average negative across subjects although not significantly different from zero (−57 ms, *t*_(11)_ = −10.950, *p* < 0.001), was also uncorrelated with PSE (subject-wise: accounted variance < 1%, *p* = 0.969, *N* = 12; subject-and-clip-wise: accounted variance < 1%, *p* = 0.658, *N* = 48; subject-and-clip-and-size-wise: accounted variance < 1%, *p* = 0.422, *N* = 144).

Finally, we found a positive significant correlation between PSE and subject age (accounted variance = 40%, *p* = 0.024, *N* = 12). However, the sample size was small and most subjects’ age was comprised between 20 and 30, thus we cautiously suggest that this result remains to be confirmed with a participants’ sample better tailored to studying age effects.

### Discussion

Experiment 1 showed a tendency toward speed underestimation, that is, a tendency toward perceiving the flow of visual events as too slow. As a consequence, observers adjusted video speed at a rate higher than the original shooting speed. The lack of a significant effect of repetition suggests that speed judgments are rather stable over time. Speed underestimation depended on video clip type, but not on display size and repetition. The largest systematic speed error was found with physical motion (C3, water waves, PSE = 25%), and smaller errors were found with human motion (C1, jumping man, PSE = 9%), ego-motion (C4, walking in the crowd, PSE = 4%), and mixed human-and-physical motion (C2, foot dribbling, PSE = 3%).

As for display size, we failed to find significant effects on PSE, either globally or with single ANOVAs. This suggests that, as far as speed is concerned, it makes no difference watching a movie on a computer monitor (the largest display size used in this experiment) or on a mobile phone (approximately the smallest display size). Clearly, it is possible that stronger manipulations turn out to be effective in modifying video speed judgments, such as immersive display viewing.

As for the duration reproduction task, we did not find evidence that duration estimation is related to speed estimation. First, at variance with speed estimation, temporal estimation did not depend on clip type. Second, no correlation was found between the performances in the two tasks, regardless of whether we considered the level of subject, subject-and-clip, or subject-and-clip-and-size level. Note that the lack of significant effects of repetition in the speed estimation task makes the presence of carry-over effects due to the fixed task sequence unlikely.

## Experiment 2

It is possible that the tendency toward speed underestimation found in Experiment 1 depended on the fact that the video clips were muted. Indeed, as anticipated, acoustic stimuli can modulate temporal processing and have a tight relation to movement (Recanzone, 2003). Therefore, Experiment 2 tested whether soundtracks can influence video speed judgments. To this aim, we asked observers to perform the same adjustment task already used in Experiment 1, with the same video clips, but this time accompanied by various soundtracks. We asked whether three acoustic manipulations (*tempo* variations of a metronome beating; *tempo* variations of a musical piece; musical pieces and white noise allegedly capable of evoking different arousal states), and in one case, volume manipulation, could modify the PSE for video speed.

### Methods

#### Participants

Twenty-one participants who did not take part in Experiment 1 (mean age = 23.76 years, 17 females) volunteered for the experiments. They had normal or corrected-to-normal vision. This study was carried out in accordance with the recommendations of San Raffaele Ethical Committee. The protocol was approved by the San Raffaele Ethical Committee. All subjects gave written informed consent in accordance with the Declaration of Helsinki.

#### Stimuli and Tasks

Stimuli and tasks were the same as in Experiment 1, except that the maximal trial duration was extended to 60 s to provide a more comfortable response temporal window (video playback was in looping mode) and that, to reduce the overall duration of the experiment, the video clip with human motion (C1) was dropped (C1 was in fact similar to C2 in terms of both content and PSE). Another difference with Experiment 1 was that at the end of each trial participants provided a confidence rating on a 1–9 scale at the end of each trial. This was a type 2 judgment, that is, a judgment of the participant’s own decision performance (targeting observer’s response, (Galvin et al., 2003) and not of the state of the external word (targeting the visual stimulus; Gregori-Grgič et al., 2011). Also, a different equipment was used (Windows 7 MiniMac, with a custom graphic board and a 21″ LG LCD monitor).

#### Soundtracks

This experiment had three experimental sessions, characterized by different soundtracks. In session 1 we used a sequence of metronome beats, with five different *tempos* (40–143 bpm), which were generated with the “TempoPerfect” Software (NCH Software). In session 2 we used a Bach piece (“*Jesus bleibet meine Freude*”, a choral from BWV 147), with five speed levels (0.67×, 0.77×, 1×, 1.3×, 1.5×, with a baseline speed of 70 bpm). Audio speed manipulations included pitch-compensation. Finally, because pure temporal manipulations may be less effective than music salience (de Bruin et al., 2015), in session 3 we used two musical pieces with an alleged relaxing or exciting effect, played at their normal speed (respectively “*Parce mihi Domine*”, a liturgical song by Cristóbal de Morales, and the pop piece “*It’s raining men*”, by Geri Halliwell), plus an additional white noise stimulus. In this session we manipulated also the soundtrack volume (two levels). The three stimuli were equalized to yield the same mean sound amplitude at each volume level (45 and 61 dB on average). In all sessions auditory stimuli were reproduced on headphones. Sound levels were measured with the “Science Journal” app for smartphones, placing the microphone close to the headphones loudspeaker. Before starting the experiment, participants were given a short familiarization session.

#### Experimental Design and Data Analysis

In session 1, we tested three video clips (C2, human-physical motion; C3, physical motion; C4, ego-motion), and five auditory conditions (different metronome *tempo*), for a total of 15 trials for each participant. In session 2 we tested the same three video clips and five auditory conditions (different musical speeds), for a total of 15 trials for each participant. In session three we tested the same three video clips, with three auditory conditions (two pieces plus white noise) and two volume levels, for a total of 18 trials for each participant. In all sessions the design involved only within-subject factors, and their presentation order was randomized. Each session lasted about 20 min. The three sessions were run in either the same or different days, according to the participants’ convenience. Participants took part in either one (*N* = 6), two (*N* = 8) or three (*N* = 7) experimental sessions, and, where possible, session order was counterbalanced across subjects so that each session had 15 subjects.

Statistical analyses were the same as those used in Experiment 1. In addition, Friedman’s test was used for confidence ratings.

### Results

In all sessions observers tended to terminate adjustments towards the middle of the trial (data not shown), suggesting that using 60 s as maximal trial duration was an over-prudent measure, and that the 30-s trial duration used in Experiment 1 was appropriate. In the following, we report the main results from the three sessions in sequential order.

#### Session 1

**Figure 4:**
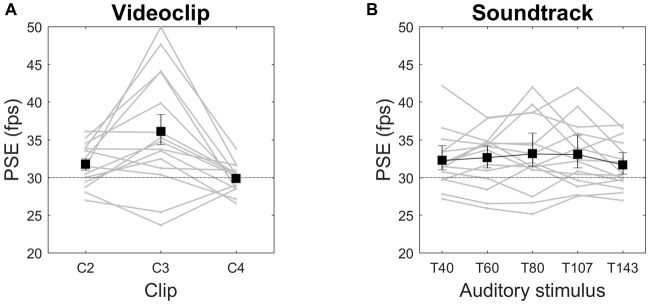
Videoclip **(A)** and Soundtrack **(B)** effects on PSE in Session 1. Means and 95% confidence intervals are reported. Gray lines are individual subjects’ data. PSE is expressed in frame per second (fps). The horizontal dotted line indicates the original video speed (30 fps).

As in Experiment 1, we found the video clip type to be the only factor capable to significantly affect PSE (*F*_(1.292,18.083)_ = 9.465, *p* = 0.004, *η*^2^ = 0.403), with speed increments of 6%, 20% and 0% for C2, C3, C4, respectively (Figure 4A). For C2 and C3, but not for C4, both the means and the lower confidence bounds remained above the objective video clip frame rate (30 fps). Median values showed a very similar pattern (speed increments of 5%, 16% and −1% for C2, C3, C4, respectively). All pairwise contrasts with the three clips were statistically significant. Neither the main effect of auditory *tempo* nor the interaction video clip × *tempo* were statistically significant, indicating that this acoustic manipulation did not influence the subjective estimation of visual speed, as also apparent from the almost constant PSE over large *tempo* variations (Figure 4B). To further examine whether speed judgments are indeed insensitive to soundtrack *tempo*, we ran three separate ANOVAs, one for each video clip data, but again no statistically significant effects emerged. As for single subjects, in nine of them (60%) PSEs were significantly above the objective video clip frame rate, in two subjects (13%) they were significantly smaller, and in four subjects (27%) confidence intervals included the 30-fps reference value (data not shown).

We found a modest but significant trial-wise correlation between the initial video clip speed and PSE (*r* = −0.135, *p* = 0.044), and a significant trial-wise correlation between total adjustment behavior and PSE (*r* = 0.134, *p* = 0.045).

The pattern of confidence ratings indicated good compliance with the task, with mean values across video clips and soundtracks ranging between 4.3 and 9. The main effect of video clip, but not of soundtrack, was statistically significant ($\chi_{\left(2\right)}^{2}$ = 8.593, *p* = 0.014, and $\chi_{\left(4\right)}^{2}$ = 5.521, *p* = 0.238, respectively).

#### Session 2

**Figure 5:**
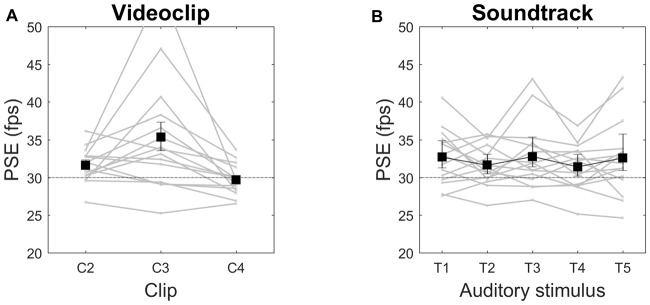
Videoclip **(A)** and Soundtrack **(B)** effects on PSE in Session 2.

In this experimental session we substantially replicated the results of Session 1 (we recall that the only experimental difference was that, instead of five *tempo* variations of a metronome beat, here we tested five *tempo* variations of a Bach’s piece). ANOVA revealed only a significant main effect of video clip (*F*_(1.066,14.922)_ = 7.421, *p* = 0.015, *η*^2^ = 0.346). All pairwise contrasts with the three clips were statistically significant. We found no statistically significant effects of soundtrack by running a separate ANOVA for each video clip data. Speed increments were 5%, 18% and −1% for C2, C3, C4, respectively (Figure 5). For C2 and C3, but not C4, the confidence intervals did not include the objective video clip frame rate. Median values showed a similar pattern (speed increments of 5%, 11% and −1% for C2, C3, C4, respectively). As for single subjects, we obtained identical proportions than in experimental session 1 (60% of observers with PSE of observer with PSE, 13% below and 27% not significantly different from 30 fps).

No significant trial-wise correlations emerged (initial video clip speed vs. PSE: *r* = 0.023, *p* = 0.734; adjustment behavior vs. PSE: *r* = −0.076, *p* = 0.256).

Mean confidence ratings ranged between 4.6 and 9. The main effect of video clip, but not of soundtrack, was statistically significant ($\chi_{\left(2\right)}^{2}$ = 8.018, *p* < 0.001, and $\chi_{\left(4\right)}^{2}$ = 2.739, *p* = 0.602, respectively).

#### Session 3

**Figure 6:**
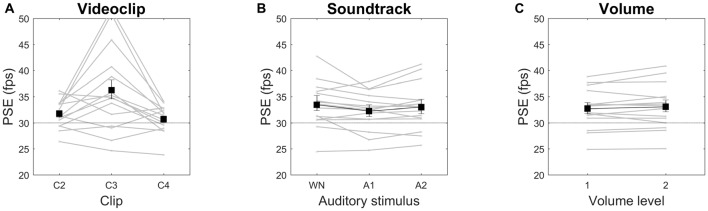
Videoclip **(A)**, Soundtrack **(B)** and Volume **(C)** effects on PSE in Session 3.

In this session we tested different soundtrack types (an allegedly relaxing piece, an allegedly arousing piece, and white noise), rather than a soundtrack with different *tempos*, and added volume manipulation. Again, we found a significant main effect of video clip (*F*_(1.091,15.279)_ = 7.777, *p* = 0.012, *η*^2^ = 0.357), and no other significant effects. All pairwise contrasts with the three clips were statistically significant. Also ANOVAs on individual video clip data failed to show significant effects. Speed increments were 6%, 18% and −1% for C2, C3, C4, respectively (Figure 6). For C2 and C3, but not C4, the confidence intervals did not include the objective video clip frame rate. Median values showed a similar pattern (speed increments of 5%, 21% and 2% for C2, C3, C4, respectively). As for single subjects, the proportions were 60% with PSE above, 20% below and 20% not significantly different from 30 fps.

PSE was significantly correlated with total adjustment behavior (*r* = 0.147, *p* = 0.015) and not with the initial video clip speed (*r* = −0.110, *p* = 0.070).

Mean confidence ratings ranged between 5.8 and 8.8. Again, the main effect of video clip was statistically significant ($\chi_{\left(2\right)}^{2}$ = 14.465, *p* = 0.018), but not that of soundtrack ($\chi_{\left(2\right)}^{2}$ = 2.159, *p* = 0.340).

### Discussion

This experiment showed that auditory manipulations failed to induce consistent PSE modifications in the visual task. This finding adds to a similar lack of effectiveness of experimental manipulations found in Experiment 1 (insensitivity of PSE to display size and repetition), and strengthens the notion that systematic, stimulus-specific errors in estimating video speed reflect rather robust biases in visual cognition. Clearly, as also noted for display size, it is always possible that stronger or different auditory manipulations turn out to be effective in modifying video speed judgments (see also “General Discussion” section).

We also found that the estimated video speed was not totally independent of the adjustment procedure, because PSE was sometimes correlated with either the initial video speed and/or observers’ adjustment behavior. This observation motivated Experiment 3.

## Experiment 3

In this experiment we aimed at replicating the speed estimation errors found in Experiments 1 and 2, but using another psychophysical method, namely, the double staircase method. Indeed, actively adjusting the video speed over time is not equivalent to making a pure perceptual judgment. In relying on the experimenter’s rather than the subject’s control, the staircase method provides a more perceptually-oriented measurement procedure (Ehrenstein and Ehrenstein, 1999). Thus, in this experiment observers simply responded whether the video clip appeared too slow or too fast. An additional though minor goal of this experiment was to ascertain whether the particularly high PSE obtained with C3 depended on some subtle characteristics of that particular video clip (e.g., anomalous frame timing, or a different view angle), as it was the only stimulus not directly shot in-house.

### Methods

#### Participants

Twenty participants (mean age = 23.00 years, 14 females) volunteered for the experiments. They had normal or corrected-to-normal vision, and were *naïve* as to the purpose of the experiment. This study was carried out in accordance with the recommendations of San Raffaele Ethical Committee. The protocol was approved by the San Raffaele Ethical Committee. All subjects gave written informed consent in accordance with the Declaration of Helsinki.

#### Stimuli and Tasks

Stimuli were prepared by randomly selecting 3 s-long portions of the two video clips used in Experiments 1 and 2 (C2 and C3). These two video clips were associated to large and small speed estimation errors, respectively, thus spanning a relatively wide perceptual range. We actually tested two versions of the stimulus that determined the highest error (C3), which allowed us to verify whether such a large speed estimation error could be due to some peculiarity of that particular video clip. The first version, called C3a, was the same that we already used in the previous experiments. The second version, called C3b, depicted the same general visual subject (water waves), but was shot with the same camera used to record video clips C1, C2 and C4 (we recall that C3 was obtained from a web video collection). Participants were divided in two groups (*N* = 10), and each group was presented the C2 video clip and either the C3a or the C3b video clip (collectively called C3).

The staircase procedure involved two series (one for C2 and the other for C3) of double (increasing and decreasing) staircases randomly interleaved, with maximum 40 trials each (for a maximum of 160 trials), targeting 50% PSE (1 up − 1 down steps), and with 16 reversals as stopping rule. The entire procedure lasted about 15 min. This experiment was conducted with the same equipment used in Experiment 2.

#### Experimental Design and Data Analysis

For each observer, PSE for each video clip was computed in two ways, respectively as the geometric mean of the last 11 responses over the two staircases, or as the video clip speed corresponding to half of the psychometric function (0.5). The psychometric function was computed through logistic fit of individual observers’ responses (General Linear Model procedure). Because PSE was practically identical when computed with the two methods, in the results we report only the PSE derived from the psychometric function This also made it possible to measure the just noticeable difference (JND), that is, the smallest discriminable speed difference as indexed by the semi-interquartile range of the psychometric function. PSE is expressed as percent departure from the original video clip frame rate and PSE as percentage of the original video clip frame rate, although for the statistical analyses the raw values were used.

The experiment was structured as a 2 × 2 design, with one within-subject (video clip) and one between-subject (group) factor. The statistical analyses were the same used in Experiment 1, except that a mixed-factors ANOVA was used. Moreover, Student’s *t*-test for independent-samples (2-tailed) assessed PSE difference between C3a and C3b.

### Results

**Figure 7:**
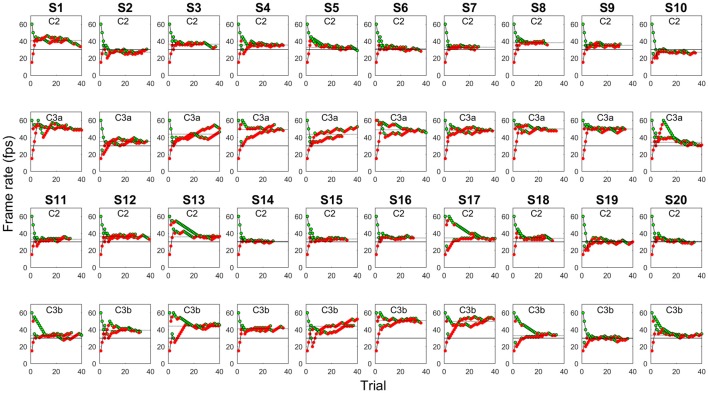
Staircase history for each observer (S#) and video clip (C#). Each data-point represents the speed at which the video is presented in each trial. Observers can respond either “too fast” (green symbols) or “too slow” (red symbols). Both the ascending and descending series are plotted. The plots indicate good response convergence towards the final estimation of the “natural” video clip speed (PSE, dotted line). Subjects 1–10 (S1–S10) were tested with the C3a video clip version, S11–S20 with the C3b version (see text). Solid line, original video clip speed (30 fps).

Observers’ responses converged rather regularly to a final plateau level. The maximum number of trials (*N* = 40) was reached 17 times, and the staircase procedure stopped on average after 33 trials (Figure 7). PSE was weakly but not significantly correlated with convergence rapidity, measured as the number of trials before the procedure stopped (accounted variance = 8.5%, *p* = 0.068).

**Figure 8:**
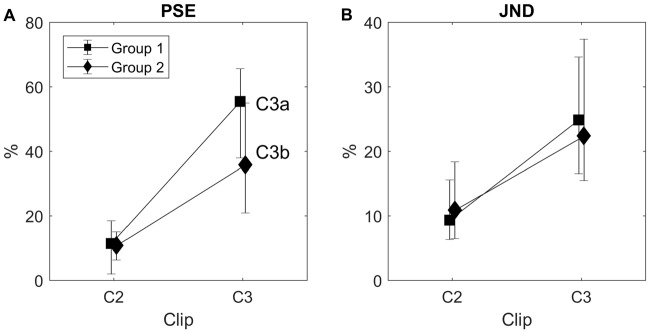
PSE **(A)** and just noticeable difference (JND) **(B)** in the two observer groups derived from the individual psychometric functions in the double staircase experiment. Video clips C3a and C3b were used in group 1 and group 2, respectively. Error bars are 95% bootstrap confidence intervals.

PSE was significantly higher than 30 fps with all visual stimuli, as attested by confidence intervals, indicating a general tendency toward speed underestimation, especially for the C3 video clips (Figure 8A). The main effect of video clip on PSE was significant (*F*_(1,18)_ = 55.171, *p* < 0.001, *η*^2^ = 0.754) and neither the main effect of group nor the interaction clip × group were significant (*F*_(1,18)_ = 1.819, *p* = 0.194, *η*^2^ = 0.092, and *F*_(1,18)_ = 4.202, *p* = 0.055, *η*^2^ = 0.189, respectively), thus suggesting that PSE was essentially modulated by the general type of visual content rather than on specific stimulus features. Indeed, despite a tendency toward larger speed underestimation with C3a than with C3b, a pairwise comparison did not reveal a significant PSE difference between these two video clips (*t*_(18)_ = 1.714, *p* = 0.104).

A similar pattern of results was found for JND (Figure 8B, main effect of video clip, *F*_(1,18)_ = 12.435, *p* = 0.002, *η*^2^ = 0.409; main effect of group, *F*_(1,18)_ = 0.014, *p* = 0.908, *η*^2^ = 0.001; interaction clip × group, *F*_(1,18)_ = 0.273, *p* = 0.608, *η*^2^ = 0.015), and PSE and JND were positively correlated (*r* = 0.465, *p* = 0.002).

### Discussion

This experiment confirmed speed underestimation found in Experiments 1 and 2, where the adjustment method was used. Moreover, we confirmed the content-specific nature of speed underestimation—constant errors with C2 and C3 showed the same pattern as in the previous experiments, PSE tended to be larger for C3. The results also excluded that the high PSE values found in Experiments 1 and 2 for C3a, which was the only video clip that was downloaded from a web collection and not directly shot, depended in any important way on some subtle clip features, as PSE did not differ significantly between C3a and C3b.

Speed sensitivity was rather poor. The JND data shown in Figure 8B indicate that changing the video speed by about 10% (for C2) or even 20% (for C3) is likely to go unnoticed. This is highly relevant when considering possible video speed optimization strategies (see the “General Discussion” section), as it defines the tolerance range around PSE within which a speed change would not be detected. Furthermore, JND data confirm that C3 is more kinematically ambiguous than C2, as it is more difficult to discriminate speed differences with C3 than with C2 video clip.

Having used the staircase method with four randomly intermingled trial sequences (2 stimuli × 2 series) made it unlikely for observers to be influenced by spurious cues related to trial sequence. Indeed, in that condition it is almost impossible for an observer to keep track of the history of each individual staircase sequence, thus excluding that a speed underestimation bias could have been introduced by converging to the speed mid-point counting the individual staircase steps. As a cons, to prevent the experimental session from lasting too long, speed judgments were based on rather short video clip pieces (3 s), thus losing large part of the visual context.

## General Discussion

In this study, we focused on the sense of speed, or subjective visual *tempo*, by measuring the perceived “right” speed of real-life scenes presented as video clips. We ran three experiments in which methods and experimental conditions were varied. The results indicate that observers tended to judge video clips to be too slow (overall, speed was underestimated by 13%), with a content-specific PSE pattern that survived across experiments (Table 1). In particular, the most kinematically ambiguous video clip, the one showing water ripples, was systematically associated with the strongest speed underestimation.

**Table 1:** Synopsis of mean points of subjective equality (PSEs) found in the three experiments.

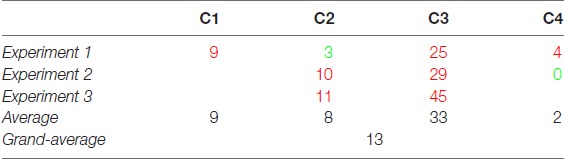

*Values are given as signed percent departures from the original video speed. In Experiment 2, data from the three sessions are pooled together, as also C3a and C3b data in Experiment 3. Statistically significant departures are indicated in red, non-significant departures in green. Black values indicate the non-weighted averages across experiments, and the non-weighted grand-average across experiments and video clips*.

PSE estimates were uncorrelated to duration estimation errors, and were not influenced by simple manipulations of either visual (display size) or auditory (soundtrack rhythm, musical tempo, musical content, volume) factors.

Speed underestimation was not an artifact. Temporal calibration was accurate, thus we can exclude temporal artifacts in video recordings. Also, the experiments were conducted on different computers, graphic cards and monitors, thus we can exclude temporal artifacts in video reproduction hardware. Furthermore, speed underestimation was found with two different psychophysical methods, adjustment and staircase, thus we can exclude a methodological artifact (although in one case staircase was associated to a higher PSE, see below).

Therefore, speed underestimation with real-life videos seems to be a genuine, replicable phenomenon, robust to experimental manipulations of display size and soundtrack. Clearly, this conclusion cannot be generalized, as PSE depended strongly on video content. It is entirely possible that certain stimuli are associated to null PSEs or even to speed overestimation (Pittenger, 1985). Only a large-scale study involving vast samples of video clips could shed light on this issue. What is important to note at this point, however, is that the original video speed was not what observers subjectively considered to be the “natural” speed. This may have important consequences for the video media industry (see below).

### Speed Biases: (Mis)interpreting the World Dynamics

The first aim of this study was to verify that different real-life visual scenes can give rise to different speed biases. It is important to remark that we did not aim at controlling video clip content, in terms of either high-level or low-level features, and stimulus selection should be considered totally arbitrary. Indeed, as anticipated, a variety of factors may be at the origin of content-specific speed biases. Thus, in the following we do not intend to definitely causally associate a particular PSE to one or another element of the video clips, but simply to examine some possible factors at play.

With real-life events we have expectations about how the world should appear. These expectations may lead to errors when reasoning about dynamical events (McCloskey and Kohl, 1983; McCloskey et al., 1983), and sometimes these errors persist even under favorable conditions such as viewing an event and not just imagining it (Kaiser et al., 1992; Crespi et al., 2012). With real-life video clips such as those used in the present study the particular appearance of objects and events may heavily influence observers’ judgments. Sometimes visual cues are well defined—for example, if the soccer ball in C2 were a volley ball, kinematic expectation would probably be different. Sometimes they may be weak—for example, wind cues in C3 may be very difficult to detect, thus giving rise to wrong kinematic expectations. In other words, the visual stimulus is matched against a set of more or less strong expectations based on previous knowledge of the world (for example a “physics engine” (Kubricht et al., 2017). With ambiguous stimuli, such as water flow, we apply a best guess as to the plausibility of the sensory input, which may lead us to interpret visual kinematics more loosely and erroneously than if stimuli had well-defined motion cues.

When human actions are at stake, speed judgments may derive not only from direct visual tuning (Runeson et al., 2000), but also from the interplay between visual and motor processes: we might be particularly sensitive to kinematic violations of observed human movements because we could rely on our own action repertoire as an internal template (de’Sperati and Stucchi, 1995, 1997, 2000; de’Sperati and Viviani, 1997; Viviani et al., 1997; Cattaneo and Rizzolatti, 2009; Gallese et al., 2009; Lacquaniti et al., 2014). This may be one reason for the smaller bias found with the C1 and C2 video clips, as compared to the C3 video clip: an excessive departure of the reproduction speed from the original video speed may easily bring about an evident violation of motor rules. It is also interesting that the speed underestimation that we have found with the C4 video clip is much smaller than that reported in a previous study in which reduced optic flow was simulated during walking (Banton et al., 2005). In our case, the filmed optic flow during a walk in a crowded street included many human actors concurrently moving in the scene, a factor that may have reduced the kinematic ambiguity of the video clip, in turn reducing PSE.

On the top of content-specific speed biases, it is possible that some general tendencies are at play. For example, a tendency toward speed underestimation may depend on the presence of a slow-motion prior, deriving from the statistical regularities of the visual world (Snowden et al., 1998; Weiss et al., 2002; Stocker and Simoncelli, 2006; Vintch and Gardner, 2014; Schütz et al., 2015). This “unfortunate quirk of our perceptual systems” (Snowden et al., 1998) has been posited to emerge under uncertainty conditions due to weak, low-contrast sensory stimuli. Indeed, videos are low-contrast, reduced versions of real scenes, thus it is plausible that this general bias could at least be weakly at play when watching video clips, with the effect of shifting PSE towards positive values. Also the tendency of the staircase method to produce higher PSEs than the adjustment method may be explained with the same mechanism: the shorter stimulus duration may have amplified the kinematic uncertainty of the video clips, in turn strengthening the slow-motion prior, and ultimately resulting in exaggerated speed underestimation. It is difficult, however, to prove the existence of a general slow-motion prior based solely on these findings. As noted by Thompson et al. (2006), the perceptual slowing observed at low contrast also admits an explanation based on cortical filters. Moreover, at high stimulus speeds, underestimation may even turn into overestimation (Thompson et al., 2006; Hammett et al., 2007).

Another possible explanation of the tendency toward speed underestimation could be that, nowadays, we live almost literally immersed in an artificial visual world, with the greatest example being probably motion pictures. Visual materials are often proposed not at their original shooting speed, but at faster, more appealing speeds. People could get used to these fast videos and even fail to notice such high speeds. Whether or not this era of compulsory video consumption has indeed changed our visual kinematics habits remains to be ascertained. However, it is remarkable that exposure to video clips of human locomotion played at an altered speed can induce adaptation in locomotion speed perception (Mather et al., 2017). The systematic speed underestimation that we have observed in this study may similarly result from adaptation to increased visual hyper-stimulation. Note that, at variance with the above-mentioned slow-motion prior, adaptation does not depend on degraded stimuli to emerge.

### Speed Biases Are Robust to Simple Audio-Visual Manipulations

Would the sense of speed change by watching a movie on a smartphone rather than on the TV, or by adding arbitrary soundtracks? Our results suggest the answer is “no” to both questions.

First, we found no influence of display size on perceived video speed, at least within a range from mobile phones to desktop monitors. This suggests that speed biases are not based on retinal stimulation but on a scaled visual representation, in turn suggesting that, at least for real-life motion pictures, speed constancy is rather good (see McKee and Smallman, 1998; Distler et al., 2000; Thornton et al., 2014). The reason why also the ego-motion video clip (C4 in the first experiment) was insensitive to display size despite the reported effect of small fields of view on optic flow (Pretto et al., 2009), could be that we did not manipulate the represented field of view but only the display size. Clearly, it is always possible that much larger or smaller displays (e.g., cinema or thumbnails, or a different visual experience such as using immersive displays), do affect speed perception.

Second, subjective visual *tempo* was not affected by soundtrack. *Prima facie*, this finding seems to be at odds with several reported effects of acoustic stimulation, including music, on temporal and visual processing (Schäfer et al., 2013). In general, it is well known, also from daily life experience, that music can induce strong emotional states and pleasant sensations (Blood and Zatorre, 2001; Zatorre, 2015), often accompanied by relevant psychophysiological changes (Krumhansl, 1997; Proverbio et al., 2015). Listening to music also influences visual perception and cognition (Jomori et al., 2013; Proverbio et al., 2015), and the rhythmic structure of auditory stimuli affects the perceived visual temporal rate (Recanzone, 2003). Moreover, music, rhythm and movements are tightly intertwined, and this relationship extends to visual metrical perception, including a specific effect of visual motion on auditory *tempo* (Su and Jonikaitis, 2011; Su and Salazar-López, 2016). However, the effects of auditory stimulation on visual motion perception are less obvious, and depend on the experimental conditions (e.g., Sekuler et al., 1997; Watanabe and Shimojo, 2001; Alais and Burr, 2004; Grassi and Casco, 2010). Indeed, coupled with our previous evidence of a lack of effect of voice-over on video speed perception (de’Sperati and Baud Bovy, 2017), the present results suggest that arbitrary soundtracks, while likely contributing a variety of efficacious emotional surrounds, do not slow-down or speed-up perceived reality in a video. One reason may be that arbitrary soundtracks do not admit an even loose audio-visual binding (Parise et al., 2013), thus dissipating the cross-modal integration potential of the auditory channel. It is possible that, similarly to motion perception with simple laboratory stimuli (Grassi and Casco, 2010), the natural, original audio recording (e.g., the sound of the ball bounces on the foot, or the sound of the water on the beach) is effective in modifying the overall speed impression when watching audio-visual clips depicting real-life scenes, as compared to muted clips. Future investigation will clarify this point. The robustness of the (biased) sense of speed to arbitrary soundtracks is nonetheless of interest given their presence in many commercial video programs and even self-made video clips.

### Probing Speed vs. Probing Time

Subjective speed perception and temporal duration estimation seem to rely on distinct mechanisms, as in the first experiment we found no correlation between PSE and either intercept or slope in the duration estimation task. The independence of speed and duration estimation may sound somewhat surprising given the link between speed and time, not only at the physical level but also as repeatedly reported in the vision science literature with both laboratory and realistic stimuli (for a review see Lacquaniti et al., 2014). One reason of this discrepancy could be that we sought to appraise the relation of estimated duration to estimated speed, rather than to the objective stimulus speed, as in previous studies (Grivel et al., 2011; Nyman et al., 2017). This suggests that the estimated duration of a motion scene is modulated by the effective but not the apparent stimulus speed, possibly because, when observer’s attention is drawn to the duration rather than the speed of an event, the subjective impression of speed may not access awareness. This would be in agreement with the rather poor speed sensitivity found in this study (JND, Experiment 3) and with a previous study on real-life video viewing that showed a worsening of speed sensitivity when speed is not explicitly attended (de’Sperati and Baud Bovy, 2017).

A more substantial difference could be that, whereas estimating duration is a sort of cumulative process where time has an extension, the sense of speed, as conceived and operationalized in this study, is about the real-time unfolding of events. As such, it is close to the notion of specious present, which William James contributed to popularizing. Thus, in targeting real-life events, our approach is unique in affording a simple measure of an otherwise complex notion of the sense of ongoing reality—even though it is a selected, artificially re-presented reality. This may be useful to gain a better insight on the mechanisms at play in those cases where the dynamics of subjective reality may change, for example during development, or in psychiatric populations, or even in patients with motor disturbances, e.g., Parkinson’s patients.

### Consequences for the Video Media and Video Game Industry

Regardless of the precise nature of the underlying mechanism, the fact that observers can systematically misjudge the speed of real-life video clips may have important consequences. The question of the “right” speed dates back to the silent film era (Brownlow, 1980). As noted by a motion picture pioneer “Theoretically, the machine speed should be the same as that of the camera which took the picture being projected, but in practice this is often far from true. The camera man grinds out a set speed, supposed to be 60 feet per minute, though often he varies widely from the mark. The actors act the scene as seems best to them, but ofttimes when the scene is projected it is discovered they have misjudged the speed of action necessary for best effect. Right here is where a really good operator who closely watches such details becomes of great value, helping out the scenes amazingly merely by changing speed on different scenes.” (Richardson, 1910). Also, “Speed is of very, very great importance and a comprehension of this fact is absolutely necessary to do really fine projection. The operator “renders” a film, if he is a real operator, exactly as does the musician render a piece of music.” (Richardson, 1911). The present study suggests that indeed videos could be optimized for speed, ideally aiming at nulling PSE for the best viewing experience or simply at saving time by slightly increasing video speed. Clearly, optimization should be implemented in a smart way, parsing the various action units present in a given video (Zacks et al., 2001; Robino et al., 2012), and applying a proper speed correction to each.

Speed optimization could be introduced in various stages of video industry, from authorship and production to post-production editing to remote control at home. In this regard, it should be borne in mind that, while the staircase method is more perceptually-oriented (Ehrenstein and Ehrenstein, 1999), the adjustment method mimics quite closely a real-life situation in which users actively set the preferred speed through remote control over a fairly long time span. An advantage of individual optimization (e.g., speed adjusted in real-time by final users through remote control) over upstream optimization (e.g., speed modulation fixed by video editors) is that represented actions can meet the eyes of the beholder. Conversely, an advantage of upstream optimization is that it relieves viewers from continuous speed control, since optimization is already implemented. For that purpose, speed optimization may rely on group testing to ascertain that speed changes remain within the subjective tolerance limits (de’Sperati and Baud Bovy, 2017). The observation that speed biases are independent of display size and soundtrack suggests that speed optimization can at least partly disregard actual audio-visual viewing conditions.

Similar considerations hold for the video games industry, where physics engines can be profitably inspired by how human cognition works (Ullman et al., 2017). An important issue in this regard will be to understand whether speed biases and sensitivity are the same under passive observation conditions, e.g., when watching movies, or when active interaction is required, e.g., when playing video games. Because interactions take place in real time, and because short-latency motor responses may by-pass the slow buildup of perceptual illusions (de’Sperati and Baud-Bovy, 2008; Bruno and Franz, 2009), it is possible that speed biases are confined to the perceptual and cognitive domains.

A final note on the frame rate video standard. All video clips used in this study were shot at 30 fps. This is currently a common standard for videos, but the 60 fps standard is going to replace it soon. While the refresh rate does not change by reproducing a motion picture at 30 or 60 fps (and in any case refresh flickering on LCD displays is virtually absent), the sensitivity of the human visual system to sampled motion suggests a relationship between frame rate and speed perception (Watson and Ahumada, 1985; Burr and Thompson, 2011). Indeed, increasing the sample frequency or introducing high temporal frequencies can modify the perceived speed of apparent motion, especially at low sampling rates and low stimulus speeds (Treue et al., 1993; Castet, 1995). However, no such speed bias has been reported with staircase motion, i.e., the type of apparent motion produced by LCD displays (Castet, 1995). Although this observation suggests null or little impact of the passage to the new frame rate standard, this point remains to be ascertained.

## Conclusion

Converging evidence from multiple experiments in this study suggests that video speed biases are a genuine and replicable phenomenon, and robust to simple visual and auditory manipulations. Speed underestimation was the most common pattern that we have found, although at present this claim cannot be generalized. Importantly, when viewing real-life video clips speed biases may be the rule rather than the exception. This finding may be exploited to develop a simple assessment tool to monitor the subjective flow of reality, and may have far-reaching implications for the world of video media and perceptual technologies.

## Author Contributions

FR acquired and analyzed the data, and contributed to writing the article. EM acquired the data. CS analyzed the data and wrote the article.

## Conflict of Interest Statement

The authors declare that the research was conducted in the absence of any commercial or financial relationships that could be construed as a potential conflict of interest.

## Footnotes

^1^Sometimes we do appreciate special effects with warped time, and also old silent films with a jerky funny appearance, but these are cases where speed alteration is manifest and clearly perceived as an anomaly.

^2^http://www.loungev.com/beach-lounge-film
